# TiLoop® Bra mesh used for immediate breast reconstruction: comparison of retropectoral and subcutaneous implant placement in a prospective single-institution series

**DOI:** 10.1007/s00238-014-1001-1

**Published:** 2014-08-03

**Authors:** Donato Casella, Marco Bernini, Lapo Bencini, Jenny Roselli, Maria Teresa Lacaria, Jacopo Martellucci, Roberto Banfi, Claudio Calabrese, Lorenzo Orzalesi

**Affiliations:** 1 Breast Unit Surgery, Oncology Department, Careggi University Hospital, L.go Brambilla 3, 50134 Florence, Italy; 2 Surgical Oncology, Oncology Department, Careggi University Hospital, L.go Brambilla 3, 50134 Florence, Italy; 3 General Surgery, Emergency Department, Careggi University Hospital, L.go Brambilla 3, 50134 Florence, Italy; 4 Drugs and Devices Service, Pharmacy Department, Careggi University Hospital, L.go Brambilla 3, 50134 Florence, Italy

**Keywords:** Titanium-coated mesh, Immediate breast reconstruction, Conservative mastectomy, Implant-based breast reconstruction, Subcutaneous implant positioning

## Abstract

**Background:**

Immediate implant reconstruction after a conservative mastectomy is an attractive option made easier by prosthetic devices. Titanized polypropylene meshes are used as a hammock to cover the lower lateral implant pole. We conducted a prospective nonrandomized single-institution study of reconstructions using titanium-coated meshes either in a standard muscular mesh pocket or in a complete subcutaneous approach. The complete subcutaneous approach means to wrap an implant with titanized mesh in order to position the implant subcutaneously and spare muscles.

**Methods:**

Between November 2011 and January 2014, we performed immediate implant breast reconstructions after conservative mastectomies using TiLoop® Bra, either with the standard retropectoral or with a prepectoral approach. Selection criteria included only women with normal Body Mass Index (BMI), no large and very ptotic breasts, no history of smoking, no diabetes, and no previous radiotherapy. We analyzed short-term outcomes of such procedures and compared the outcomes to evaluate implant losses and surgical complications.

**Results:**

A total of 73 mastectomies were performed. Group 1 comprised 29 women, 5 bilateral procedures, 34 reconstructions, using the standard muscular mesh pocket. Group 2 comprised 34 women, 5 bilateral procedures, 39 reconstructions with the prepectoral subcutaneous technique. Baseline and oncologic characteristics were homogeneous between the two groups. After a median follow-up period of 13 and 12 months, respectively, no implant losses were recorded in group 1, and one implant loss was recorded in group 2. We registered three surgical complications in group 1 and two surgical complications in group 2.

**Conclusions:**

Titanium-coated polypropylene meshes, as a tool for immediate definitive implant breast reconstruction, resulted as safe and effective in a short-term analysis, both for a retropectoral and a totally subcutaneous implant placement. Long-term results are forthcoming. A strict selection is mandatory to achieve optimal results.

Level of Evidence: Level II, therapeutic study.

## Introduction

Skin-sparing and nipple-sparing mastectomies (SSM, NSM), which are defined conservative mastectomies [1], have changed perspectives and possibilities of breast reconstruction.

Implant-based breast reconstruction (IBBR) is the most frequently used technique worldwide. This is true both in USA [2] and in Europe. The adoption of a delayed procedure using a tissue expander (TE) before the definitive implant is the easiest and the most common solution. Nonetheless, an immediate IBBR with a definitive implant is sometimes accomplished. A full muscular pocket is not always feasible, particularly for large implants. The use of prosthetic devices allows surgeons to perform a mastectomy with immediate definitive implant placement and as a one-step procedure with greater frequency than in the past.

Many solutions have been developed in the past years, spanning from biological acellular dermal matrix (ADM) to meshes of diverse prosthetic materials. Every device is usually adopted as a sling. The technical employment is based on the principle of implant lower lateral pole coverage in a hammock-like fashion after major pectoralis muscle detachment. ADMs have been used for many years as compared to prosthetic meshes and are approved both in Europe and in USA; a large amount of data are present in literature on their use and results [3–12]. ADMs should be considered, to date, the gold standard for soft tissue replacement in immediate IBBR. Notwithstanding their utility and importance, ADMs have shown, not always at a statistically significant level, a trend toward a higher complication rate when compared to standard whole muscular pocket implant reconstruction, particularly infections and seromas [13–18].

Titanium-coated polypropylene mesh (TCPM), TiLoop® Bra (TiLOOP® Bra, pfm medical, Cologne, Germany), has been approved for use in breast surgery since 2008 in Europe. It is a very useful prosthetic mesh, proven to be safe and effective in IBBR [19]. In the largest series present in literature to date regarding TiLoop® in IBBR [20], with more than 200 cases, the major complication rate is 13.4 %, minor complication rate is 15.6 %, and implant loss rate is 8.7 %.

After having used ADM and TiLoop® meshes alternatively for some years, we designed and started a single-institution nonrandomized prospective study on TiLoop® Bra. The aim of the present study was to compare the TiLoop® Bra use in IBBR either in its standard sling fashion for a retropectoral implant positioning or in an implant full coverage for a prepectoral totally subcutaneous position. This type of approach comprises the only use of mesh without any muscle dissection and submuscular pocket.

## Patients and methods

### Study design

In 2011, we designed a prospective, nonrandomized clinical study of immediate IBBR by means of a titanium-coated mesh used in two different ways. Patients scheduled at our institution for conservative mastectomies, either NSM or SSM, followed by immediate definitive prosthetic reconstruction using TCPM, specifically the TiLoop® Bra, would have been entered into a computer-based database and followed up every 2 months after surgery. The two types of reconstruction, e.g., the mesh use and implant positioning, adopted in our Breast Unit were not randomized and were left at the single surgeon preference after a thorough conversation with each patient. Cases with the traditional retropectoral muscular mesh implant coverage made up the first group, group 1, while those with a mesh-only coverage, prepectoral and totally subcutaneous, comprised the second one, group 2. Patients were included in the study only if the following selection criteria were met: age less than 80 years, normal BMI (range 18.5–24.9), small-medium size breasts, and ptosis grade of the first and second degree according to the three-tier Regnault ptosis scale [21]. Exclusion criteria were previous breast surgery, T4 and metastatic cancers, refusal to sign the consent, comorbidities (diabetes, renal failure, congestive heart failure, cardiovascular diseases including hypertension, pulmonary diseases, chronic hepatic diseases, and metabolic diseases), smoking, and previous radiotherapy on the chest wall. A detailed informed consent including a technical surgery description and complication information had to be signed by every woman. The prospective digital database of the study cases includes all baseline characteristics, oncologic data, surgical technical information, and short-term outcomes. The database also includes postoperative complications and follow-up outpatient visits. Short-term outcomes were classified as follows: IBBR failure (e.g., implant removal) and surgical complications, namely, skin-nipple necrosis, seroma, wound dehiscence, wound or skin flap infection, hematoma, and atopic versus graft reaction. The study was conducted in accordance with the Declaration of Helsinki, and after, the Hospital Ethical Committee reviewed and approved the study protocol and all its related documentation.

A 2-year enrollment period was initially considered in the study protocol for the first analysis of results with a prediction of approximately 30 cases per group. Two independent physicians were in charge of the study control with periodic evaluation of interim results. The study would have been stopped in a case of statistically significant adverse events. Case data (patients’ baseline characteristics, tumor staging, biomolecular details, and short-term outcomes of the two groups) were intended to be compared considering the total count of mastectomy procedures rather than the number of women operated on.

### Surgical technique

#### Group 1

After mastectomy completion, once skin flaps were judged adequate, pectoralis major muscle dissection and detachment from chest wall were performed. Serratus muscle was spared and not used. A retropectoral pocket was hence created. An adequately selected implant was put in place, and a TCPM (TiLoop® Bra, Large Extralight Sheet) was then employed as a hammock to cover the lower lateral pole of the implant. Two Jackson-Pratt (JP) drains were left in place, one under the combined pocket and the other in a more superficial subcutaneous site, and removed only if less than 40 cc/24 h were collected after two consecutive days. An additional drain was employed dependent on axilla management. An antibiotic treatment was started intravenously 30 min before incision and then given per os for 4 postoperative days. A physical rehabilitation therapist followed the patient from postoperative day 1 as needed for exercise and movement suggestions.

#### Group 2

**Fig. 1:**
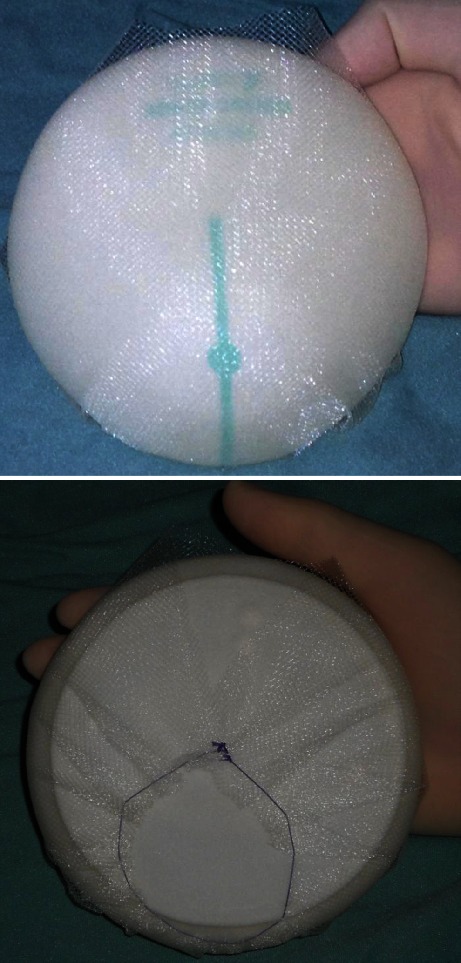
Complete implant wrapping with TiLoop® Bra for subcutaneous placement

An identical approach was carried out for the second group of patients except for the TCPM and muscular management. Once skin-fat flaps were considered adequate, a TiLoop® Bra mesh bag was adjusted around the implant but without cutting the mesh itself, in order to prevent sharp edges or spikes. Using reabsorbable sutures, a TiLoop®sheet (TiLoop® Bra, Large Extralight Sheet) was folded onto itself to create a bag which eventually functioned as a pocket for a breast implant (Fig. 1). In the case of larger implants, two TCPM sheets were used and stitched together. The TCPM bag, with the implant inside, was then placed in a prepectoral totally subcutaneous position. Medial and lateral borders were usually secured to the muscular fascia with few interrupted reabsorbable sutures. Only one JP drain was left, and no physical therapist consult was normally required.

### Statistical analysis

Analysis of recorded data was done using the IBM SPSS Statistics software (SPSS version 19, SPSS Inc., Chicago, IL, USA). Significance of differences was calculated using nonparametric tests. Mann-Whitney *U* test was used for continuous variables, while the chi-square test or Fisher’s exact test was used where appropriate. A two-tailed *p* value inferior to 0.05 was considered statistically significant.

## Results

Between November 2011 and January 2014, a total of 73 mastectomies were enrolled in the study. Ten were bilateral procedures, meaning that 63 women were submitted to this type of surgery. Thirty-four immediate IBBR performed on 29 women, five bilateral, were accomplished with a retropectoral implant in group 1 (G1). A total count of 39 immediate reconstructions with a TiLoop® Bra mesh total implant coverage and without any muscular dissection were performed on 34 women, five bilateral mastectomies in group 2 (G2). Surgery was performed for oncologic reasons, either for malignant diseases or as prophylaxis in all cases. No statistically significant difference was present in terms of patients’ age and BMI. Oncologic variables were similar as well as the proportion of SSM and NSM in G1 and in G2. Neoadjuvant therapy was performed only in one patient in G1 and in four cases in G2. Median follow-up and range were 13 (3–29) months for G1 patients and 12 (3–27) months for G2. Differences were not significant. Overall complications rate was comparable. Among the first group of patients, we had no failures (removal of implant) while one case was registered in G2. We recorded three cases of complications in G1: two skin flap infections and one wound dehiscence. Two surgical complications, besides the one implant loss, were reported in G2: one nipple partial necrosis and one case of hematoma. All baseline, oncologic, and short-term outcome data are listed in Table 1 for both groups.

**Table 1:** Comparison of patients’ characteristics, oncologic data, and complications between the two groups

	Muscular mesh pocket G1	Mash bag pocket G2	*p* value
*N* cases (%)	34 (47)	39 (53)	
Age median (range)	51 (27–69)	47 (31–76)	0.12
BMI median (range)	23 (19–25)	23 (19–24)	0.11
Intervention *N* cases (%)			
SSM	5 (15)	3 (8)	
NSM	29 (85)	36 (92)	
			0.46
Pathology *N* cases (%)			
pT0	4 (12)	11 (28)	
pTis	7 (20)	7 (18)	
pT1mic	3 (9)	0 (0)	
pT1a	0 (0)	4 (10)	
pT1b	5 (15)	3 (8)	
pT1c	11 (32)	11 (28)	
pT2	3 (9)	3 (8)	
pT3	1 (3)	0 (0)	
			0.12
pN 0	25 (73)	26 (66)	
pN 1mi	2 (6)	1 (3)	
pN 1a	4 (12)	7 (18)	
pN 2a	2 (6)	4 (10)	
pN 3a	1 (3)	1 (3)	
			0.82
Vascular invasion (+)	5 (15)	5 (13)	0.54
ER+	25 (73)	25 (64)	0.27
PgR+	23 (68)	21 (54)	0.16
Ki67 > 16 %	19 (56)	16 (41)	0.15
Staging			
0	11 (32)	17 (43)	
IA	14 (41)	10 (25)	
IB	1 (3)	1 (3)	
IIA	5 (15)	5 (13)	
IIB	1 (3)	1 (3)	
IIIA	1 (3)	4 (10)	
IIIB	0 (0)	0 (0)	
IIIC	1 (3)	1 (3)	
			0.75
Neoadjuvant therapy: *N* cases (%)	1 (3)	4 (10)	0.36
Mortality *N* cases (%)	0 (0)	0 (0)	1.00
Overall complications *N* cases (%)	3 (9)	3 (8)	0.59
Implant loss	0 (0)	1 (3)	0.54
Skin-nipple necrosis	0 (0)	1 (3)	0.54
Seroma	0(0)	0(0)	1.00
Wound dehiscence	1 (3)	0 (0)	0.47
Wound-skin infection	2 (6)	0 (0)	0.22
Hematoma	0(0)	1(3)	0.54
Atopic reaction versus prosthesis	0 (0)	0 (0)	1.00
Reoperation	0 (0)	1 (3)	0.54
Months of FU median (range)	13 (3–29)	12 (3–27)	0.43

Failure rate and complication rate were not statistically different among the two groups.

## Discussion

Silicone implants are widely used worldwide, being IBBR by far the preferred way of breast reconstructive surgery after mastectomy. Prosthesis coverage by a whole muscular pocket in order to prevent skin flap/wound dehiscence and implant exposure has been the safest choice for years until recently. With the introduction of soft tissue replacement devices, either biological or synthetic, a combined muscular prosthetic pocket has become an attractive option. Such devices give the opportunity of enlarging the pocket and thus performing a definitive IBBR with a good volume silicone prosthesis in a one-step fashion. We started our experience with ADM and TiLoop® Bra meshes many years ago and eventually decided to start a prospective study with two different possibilities of TiLoop® adoption in November 2011. Titanized mesh can be used either as a sling to cover the lower lateral implant pole, which is cranially placed in a standard retropectoral position or as a whole implant wrapping device for a prepectoral totally subcutaneous implant position. There are few publications about breast implant subcutaneous positioning [22–24] and no publications with TCPM. The rationale for our prepectoral TiLoop® Bra use was simply based on the premise that TCPM shows significant minor flogistic histopathological reactions when compared to other polypropylene meshes [25–27]. TCPM proved to be safe when placed under the mastectomy cutaneous flap in the lower lateral definitive prosthesis pole [19, 20], and in addition, meshes are traditionally placed where mechanical stress forces are highest. Moreover, TiLoop® Bra use could have a role in the reduction of capsular contracture as well [28]. Therefore, we assumed that a mesh coverage could be extended to all the implant surface without muscle detachment and in a prepectoral position. In our analysis of results, cases were considered as the overall number of procedures and not simply the number of patients, thus entailing that a bilateral intervention was like two cases, both in terms of characteristics and complications. Results of our prospective series are encouraging with comparable failure and complication rate and in line with the current literature on this topic. We recorded just one implant loss due to a large flap necrosis in G2 (prepectoral implants). This case required implant and mesh removal 7 days after mastectomy. A TE was hence placed in a full muscular pocket under pectoralis major and serratus, which were not previously dissected and therefore still viable and safe. A latissimus dorsi flap was used to replace the skin flap necrosis area. Complication rate was similar and very limited. Except for the cases of implant removal and hematoma in G2, which required a second intervention, all the other complications were treated conservatively with antibiotics and minor surgical debridement, healing always within a month period. Flap bruise and ecchymosis not requiring invasive procedures or reintervention were not considered hematomas. We did not register any seroma, meaning a long-lasting serous fluid drainage or collection needing aspiration from the mastectomy site. Seromas and infections are drawbacks of ADM use, highlighted in published studies on this topic [13–16], both ranging from 0 to 9 %, in direct to implant reconstruction as reported by a quite recent literature review [16]. We have been using ADM for years, and we completed our learning curve in soft tissue replacement devices using ADM.

Our limited number of surgical complications might be explained by the quite restrictive selection criteria of the study design and by our previous experience with ADM.

We must acknowledge that our results in this first analysis are limited to short-term outcomes and, therefore, surgical complications. Obviously, we did not include any of those complications that need a second-step plastic revision surgery, such as capsular contracture, palpable implant signs, visible implant’s profile or poor cosmetic results, which require a longer period of observation and will be part of a future evaluation.

Our rates are lower than those reported in the largest series on TiLoop® use in breast reconstruction [20]. Lower rates could be explained by the different selection criteria. Specifically, the selection criteria in the aforementioned multicentric study was more diverse in patient characteristics including reconstructions after modified radical mastectomy and delayed IBBR with TE use.

The criteria in our study limited the technique to circumscribed cases. From our experience, we can possibly assume that a subcutaneous implant within a mesh bag is considerably safe in normal weight, nonsmoking, nondiabetic, small-medium breast-sized women. On the other hand, there could be adverse risk to thin subjects with large and ptotic breasts and patients with previous breast surgery, irradiation or risk factors such as obesity, history of smoking, diabetes, and other comorbidities. A hypothetical advantage in terms of pain and functional recovery is implied in the mesh bag technique although it was not part of the evaluation of the present study.

In our series, a postoperative radiation therapy was adopted only in cases of more than three positive lymph nodes, and it was limited to axillary and clavicular stations, according to our regional and institutional multi-disciplinary guidelines. Thus, no thoracic wall postoperative radiation therapy was adopted in any of our cases.

Other limits of our study are the following: A scientific and reproducible functional test was not performed, and the surgical site infection rate cannot be considered completely reliable in every case since a minimum follow-up of 1 year was not completed for all patients, in accordance to Centers for Disease Control (CDC) 2010 guidelines for breast implant surgery [29]. Further follow-up, analyses of long-term cosmetic results, and evaluation of pain, functional recovery, and possibly costs would definitely confirm usefulness of this approach and use of TCPM.

In conclusion, we can state that a titanium-coated polypropylene mesh used as a tool for immediate definitive IBBR resulted, in the short term, to be safe and effective both for a retropectoral and totally subcutaneous implant placement. Long-term results are forthcoming. Strict adherence to selection criteria is mandatory to achieve optimal results.

## References

[1] Veronesi U, Stafyla V, Petit JY (2012). Conservative mastectomy: extending the idea of breast conservation. Lancet Oncol.

[2] Jagsi R, Jiang J, Momoh AO (2014). Trends and variation in use of breast reconstruction in patients with breast cancer undergoing mastectomy in the United States. J Clin Oncol.

[3] Weichman KE, Wilson SC, Weinstein AL (2012). The use of acellular dermal matrix in immediate two stage tissue expander breast reconstruction. Plast Reconstr Surg.

[4] Peled AW, Foster RD, Stover AC (2012). Outcomes after total skin-sparing mastectomy and immediate reconstruction in 657 breasts. Ann Surg Oncol.

[5] Peled AW, Foster RD, Garwood ER (2012). The effects of acellular dermal matrix in expander-implant breast reconstruction after total skin-sparing mastectomy: results of a prospective practice improvement study. Plast Reconstr Surg.

[6] Hill JL, Wong L, Kemper P (2012). Infectious complications associated with the use of acellular dermal matrix in implant-based bilateral breast reconstruction. Ann Plast Surg.

[7] Newman MI, Swartz KA, Samson MC (2011). The true incidence of near-term postoperative complications in prosthetic breast reconstruction utilizing human acellular dermal matrices: a meta-analysis. Aesthetic Plast Surg.

[8] Colwell AS, Damjanovic B, Zahedi B (2011). Retrospective review of 331 consecutive immediate single-stage implant reconstructions with acellular dermal matrix: indications, complications, trends and costs. Plast Reconstr Surg.

[9] Chun YS, Verma K, Rosen H (2010). Implant-based breast reconstruction using acellular dermal matrix and the risk of postoperative complications. Plast Reconstr Surg.

[10] Antony AK, Mc Carthy CM, Cordeiro PG (2010). Acellular human dermis implantation in 153 immediate two-stage tissue expander breast recontructions: determining the incidence and significant predictors of complications. Plast Recontr Surg.

[11] Breuing KH, Colwell AS (2009). Immediate breast tissue expander implant reconstructions with inferolateral AlloDerm hammock and postoperative radiation: a preliminary report. Eplasty.

[12] Becker S, Saint-Cyr M, Wong C (2009). AlloDerm versus DermaMatrix in immediate expander-based breast recontruction: a preliminary comparison of complication profiles and material complicance. Plast Reconstr Surg.

[13] Michelotti BF, Brooke S, Mesa J (2013). Analysis of clinically sgnificant seroma formation in breast reconstruction using acellular dermal grafts. Ann Plast Surg.

[14] Ibrahim AM, Ayeni OA, Hughes KB (2013). Acellular dermal matrices in breast surgery: a comprehensive review. Ann Plast Surg.

[15] Brziezienski MA, Jarell JA (2013). 4 th, Mooty RC et al. Classification and management of seromas in immediate breast reconstruction using the tissue expander and acellular dermal matrix technique. Ann Plast Surg.

[16] Israeli R (2012). Complications of acellular dermal matrices in breast surgery. Plast Reconstr Surg.

[17] Nahabedian MY (2012). Acellular dermal matrices in primary breast reconstruction: principles, concepts and indications. Plast Reconstr Surg.

[18] Ganske I, Verma K, Rosen H (2013). Minimizing complications with the use of acellular dermal matrix for immediate implant-based breast reconstruction. Ann Plast Surg.

[19] Dieterich M, Reimer T, Dieterich H (2012). A short-term follow-up of implant based breast reconstruction using a titanium-coated polypropylene mesh (TiLoop® Bra). Eur J Surg Oncol.

[20] Dieterich M, Paepke S, Zwiefel K (2013). Implant-based breast reconstruction using a titanium-coated polypropylene mesh (TiLOOP® Bra): a multicenter study of 231 cases. Plast Reconstr Surg.

[21] Regnault P (1976). Breast ptosis: definition and treatment. Clin Plast Surg.

[22] Benediktsson K, Perbeck L (2006). Capsular contracture around saline-filled and textured subcutaneously-placed implants in irradiated and non-irradiated breast cancer patients: five years of monitoring of a prospective trial. J Plast Reconstr Aesthet Surg.

[23] Cheng A, Lakhiani C, Saint-Cyr M (2013). Treatment of capsular contracture using complete implant coverage by acellular dermal matrix: a novel technique. Plast Reconstr Surg.

[24] Schmitz M, Bertram M, Kneser U, Keller AK, Horch RE (2013). Experimental total wrapping of breast implants with acellular dermal matrix: a preventive tool against capsular contracture in breast surgery?. J Plast Reconstr Aesthet Surg.

[25] Scheidbach H, Tamme C, Tannapfel A (2004). In vivo studies comparing the in vivo biocompatibility of various polypropylene meshes and their handling properties during endoscopic total extraperitoneal (TEP) patchplasty: an experimental study in pigs. Surg Endosc.

[26] Scheidbach H, Tannapfel A, Schmidt U (2004). Influence of titanium coating on the biocompatibility of a heavyweight polypropylene mesh. An animal experimental model. Eur Surg Res.

[27] Schug-Pass C, Tamme C, Tannapfel A (2006). A lightweight polypropylene mesh (TiMesh) for laparoscopic intraperitoneal repair of abdominal wall hernias: comparison of biocompatibility with the DualMesh in an experimental study using the porcine model. Surg Endosc.

[28] Bergmann PA, Becker B, Mauss KL (2014). Titanium-coated polypropylene mesh (TiLoop Bra®)—an effective prevention for capsular contracture?. Eur J Plast Surg.

[29] Degnim AC, Throckmorton AD, Boostrom SY (2012). Surgical site infection after breast surgery: impact of 2010 CDC reporting guidelines. Ann Surg Oncol.

